# Breath detection algorithms affect multiple-breath washout outcomes in pre-school and school age children

**DOI:** 10.1371/journal.pone.0275866

**Published:** 2022-10-14

**Authors:** Marc-Alexander Oestreich, Florian Wyler, Bettina S. Frauchiger, Philipp Latzin, Kathryn A. Ramsey

**Affiliations:** 1 Division of Paediatric Respiratory Medicine and Allergology, Department of Paediatrics, Inselspital, Bern University Hospital, University of Bern, Bern, Switzerland; 2 Graduate School for Health Sciences, University of Bern, Bern, Switzerland; China University of Mining and Technology, CHINA

## Abstract

**Background:**

Accurate breath detection is essential for the computation of outcomes in the multiple-breath washout (MBW) technique. This is particularly important in young children, where irregular breathing is common, and the designation of inspirations and expirations can be challenging.

**Aim:**

To investigate differences between a commercial and a novel breath-detection algorithm and to characterize effects on MBW outcomes in children.

**Methods:**

We replicated the signal processing and algorithms used in Spiroware software (v3.3.1, Eco Medics AG). We developed a novel breath detection algorithm (custom) and compared it to Spiroware using 2,455 nitrogen (N_2_) and 325 sulfur hexafluoride (SF_6_) trials collected in infants, children, and adolescents.

**Results:**

In 83% of N_2_ and 32% of SF_6_ trials, the Spiroware breath detection algorithm rejected breaths and did not use them for the calculation of MBW outcomes. Our custom breath detection algorithm determines inspirations and expirations based on flow reversal and corresponding CO_2_ elevations, and uses all breaths for data analysis. In trials with regular tidal breathing, there were no differences in outcomes between algorithms. However, in 10% of pre-school children tests the number of breaths detected differed by more than 10% and the commercial algorithm underestimated the lung clearance index by up to 21%.

**Conclusion:**

Accurate breath detection is challenging in young children. As the MBW technique relies on the cumulative analysis of all washout breaths, the rejection of breaths should be limited. We provide an improved algorithm that accurately detects breaths based on both flow reversal and CO_2_ concentration.

## Introduction

Over recent years, the multiple-breath washout (MBW) technique has emerged as a sensitive method to identify and monitor early changes in ventilation inhomogeneity, particularly in children with cystic fibrosis (CF) [1]. During tidal breathing, the MBW test assesses ventilation distribution and lung volumes by the washout of an inert tracer gas (suflur hexafluoride or nitrogen) from the lungs [2]. Its primary outcome, the lung clearance index (LCI), is sensitive to changes in clinical status of patients with CF (e.g. lower respiratory tract infections or pulmonary exacerbations) [3–5] and is associated with the extent of structural lung disease on chest CT and ventilation or perfusion impairment on functional MRI [6, 7].

Accurate breath detection (i.e., deciding where an inspiration ends and the consecutive expiration begins and vice versa) is essential for the MBW technique, as the computation of the main outcomes (functional residual capacity (FRC) and LCI) requires end‐tidal tracer-gas concentrations [8]. Adults are generally able to perform relaxed or fixed volume tidal breathing during the test and automated breath-detection algorithms can accurately detect breath starts and ends. In young children, however, irregular breathing with pauses, variable respiratory flows, and low respiratory volumes is common [9]. Irregular breathing patterns can challenge automated breath-detection algorithms, because the analysis of flow and additional signals (e.g. carbon dioxide) depends on predetermined thresholds [10]. It is also challenging for users to assess quality and interpret the results from these tests [11].

Recent ATS/ERS statements emphasize the importance of regular tidal volume (V_T_), stable end-expiratory lung volume (EELV), and importantly, the transparency of breath detection algorithms to the operator [8, 9]. However, breath-detection algorithms in commercially available devices (e.g. Exhalyzer D/Spiroware setup, Eco Medics AG, Duernten, Switzerland) are not well documented or described in the operators manual [12], and it remains unclear why certain breaths are rejected from the analysis.

In this study, we aimed to i) investigate the breath detection algorithm used in the Exhalyzer D MBW system with cross-sensitivity error corrected analysis software (Spiroware^®^ 3.3.1), the most commonly used nitrogen MBW device used in clinical trials, ii) develop a novel algorithm for automated breath detection based on flow and CO_2_ signals, and iii) compare the algorithm used in the commercial software and a recently proposed alternative to our custom algorithm.

## Methods

### Study design and population

This was a retrospective observational study on breath-detection algorithms to detect breath ends in MBW measurements. We included paediatric MBW data from healthy infants (Basel-Bern Infant Lung Development (BILD) cohort [13]), and patients diagnosed with CF from infancy to adolescence attending their regular 3‐monthly outpatient clinic [14] or study visits [15] (Swiss Cystic Fibrosis Infant Lung Development (SCILD) cohort). The Ethics Committee of the Canton of Bern, Switzerland approved the study protocol (B2019-01072, PB_2017–02139) and parents gave written consent. We included raw MBW data (2699 trials) from 1045 test occasions in Bern between January 2014 and September 2020. Study participants were categorized in age groups as infants (0 to 2 years), pre-school age (>2 to 6 years), school age (>6 to 11 years) or adolescents (>12 years; Table 1 and S1 Fig).

**Table 1:** Study population characteristics. Displayed as mean (SD) if not indicated otherwise. A-Files are text files that contain raw data on flow, oxygen, carbon dioxide, and molar mass signals of a multiple-breath washout test. Age on test date as mean (min; max), total breaths detected by Spiroware 3.3.1 (min; max).

	Infancy	Pre-school	School-age	Adolescence
**Subjects** (female) [n]	82 (41)	11 (3)	31 (12)	23 (9)
**Visits** [n]	100	96	395	453
**A-Files** [n]	319	240	1041	1093
**Age** [years]	0.4 (0.1; 1.3)	5.2 (4.0; 6.0)	9.0 (6.0; 12.0)	14.6 (12.0; 18.8)
**Weight** [kg]	5.9 (2.3)	19.1 (2.0)	28.1 (6.1)	49.2 (10.4)
**Height** [cm]	61.2 (9.6)	111.5 (4.4)	131.5 (10.0)	157.9 (11.6)
**Total breaths detected** [min; max]	54; 232	21; 101	20; 228	16; 232

### Data analysis

Raw data consisted of Spiroware A-Files, which are text files that contain raw flow, oxygen (O_2_), carbon dioxide (CO_2_) and molar mass (MM) signals sampled at 200 Hz. Data were gathered using a mainstream ultrasonic flowmeter (Exhalyzer D, Spiroware, Eco Medics AG, Duernten, Switzerland) according to current consensus guidelines and quality controlled as described previously [11, 14, 16].

We developed LungSim 1.01, a custom Python script, to replicate the signal processing and outcome calculation of MBW trials used in Spiroware analysis software (v3.3.1, Eco Medics AG, Duernten, Switzerland) [17]. LungSim enabled us to perform all signal processing steps as previously described [16] (e.g. ambient temperature and pressure (ATP) correction, dynamic delay correction, body temperature, pressure, saturated with water vapor (BTPS) correction, filtering, cross-talk correction, breath detection, and drift correction) with individual breath detection algorithms and subsequent computation of breath tables and MBW outcomes. A detailed summary of the agreement between Spiroware and LungSim is provided in the S1 File.

### Breath detection algorithms

**Fig 1:**
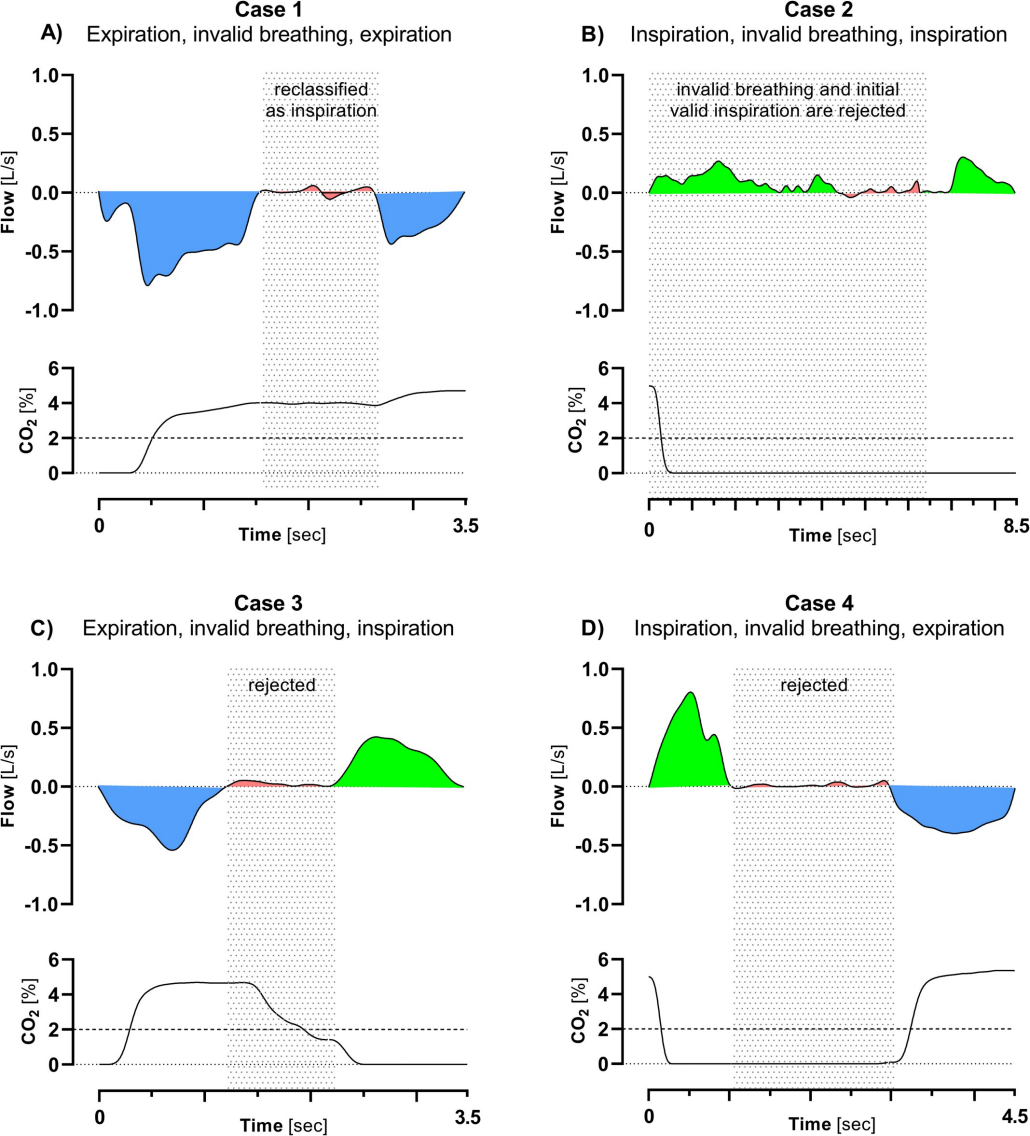
Invalid breathing as detected by Spiroware analysis software. Shown are flow and carbon dioxide traces of intervals of inspirations, expirations, and invalid breathing before re-classification (A) or rejection (B, C, D). The shaded areas indicate reclassified intervals. Blue area: expiration; red area: invalid breathing; green area: inspiration.

- Spiroware 3.3.1 (SPW; reference standard)
  Zero-crossings in the mainstream flow signal are defined as a time interval where the airflow changes its direction (from plus to minus for inspirations or vice versa for expirations). These flow changes are numbered from 1 to *N*, with *N* being the total number of zero-crossings in a MBW trial. The volume of each inspiration and expiration is calculated by integration of the flow curve over the corresponding time between zero crossings. Next, a set of conditions are applied under which raw data might be rejected: First, MBW trials always start with a valid inspiration (insp) followed by a valid expiration (exp) where “valid” is defined by a minimal volume criterion (stored in the A-File header as variable “*VS*”). Second, a breath always starts with a valid inspiration and ends with a valid expiration, applying specific principles. For an interval of:
  - Expiration, invalid breathing, expiration: the invalid breathing is kept and classified as one inspiration (regardless of whether there are one or more zero crossings between the two valid expirations).
  - Inspiration, invalid breathing, inspiration: the invalid breathing as well as the initial valid inspiration are rejected.
  - Expiration, invalid breathing, inspiration: the invalid breathing is discarded.
  - Inspiration, invalid breathing, expiration: the invalid breathing is discarded.
  Data within discarded breaths is deleted from the signals, and not taken into account for outcome computation (Fig 1).
- Custom
  Based on previously proposed algorithms, we developed a custom breath detection algorithm [10, 18]. Similar to Spiroware, zero-crossings in the mainstream flow signal are detected, numbered, and the corresponding CO_2_ concentration is calculated. Valid expirations start with a CO_2_ concentration close to zero, and end with one above a threshold of 2%. Based on this assumption, breaths are identified using the synchronized CO_2_-signal:
  - For expirations: in a sequence of zero-crossings which all have a CO_2_ concentration below the threshold of 2%, and which is followed by a zero-crossing with a CO_2_-concentration above 2%, only the last zero-crossing below 2% is identified as a valid start to an expiration.
  - For inspirations: in a sequence of zero-crossings with CO_2_ concentrations above 2%, followed by a zero-crossing below 2%, only the last zero-crossing above 2% is identified as a valid start to an inspiration.

While the custom and the recently described Horáček breath detection algorithm both rely on the analysis of flow and corresponding CO_2_ concentrations, the custom algorithm i) does not include a lower threshold for CO_2_, ii) does not include any threshold for the volume ratio (V_insp_ / V_exp_) between two intervals, and iii) does not rely on thresholds for the flow signal.

### Comparison of algorithms

Raw data was analyzed in LungSim:

1. with the Spiroware breath detection algorithm (SPW)
2. with our custom breath detection algorithm (custom)
3. with a previously published Horáček breath detection algorithm (HOR) (details provided in the S1 File).

### Statistical analysis

Primary outcomes were differences in the total number of detected breaths and resulting differences in main MBW outcomes (cumulative expired volume (CEV), functional residual capacity (FRC), and lung clearance index (LCI)) between breath detection algorithms. Differences were characterized using paired t-tests. Intergroup differences were compared using unpaired t-tests. Statistical analysis was performed using STATA 16.1 (StataCorp, College Station, USA) and GraphPad Prism 8 (GraphPad Software, San Diego, USA). A p≤0.05 was considered significant.

## Results

### Agreement between the Spiroware and custom breath-detection algorithm

There was a systematic difference in the number of total breaths detected for both the N_2_ and SF_6_ trials between the Spiroware and custom breath-detection algorithm. The majority of trials (81%) differed by two breaths (mean (SD) 1.8 (1.6) breaths, p<0.001), with Spiroware detecting more breaths than the custom algorithm in 98% of trials. The maximum difference in breaths detected between the Spiroware and custom algorithms was -22.6% (19 breaths out of 84 detected by Spiroware) in a N_2_-MBW trial and -20.6% (33 breaths out of 160 detected by Spiroware) in a SF_6_-MBW trial.

**Fig 2:**
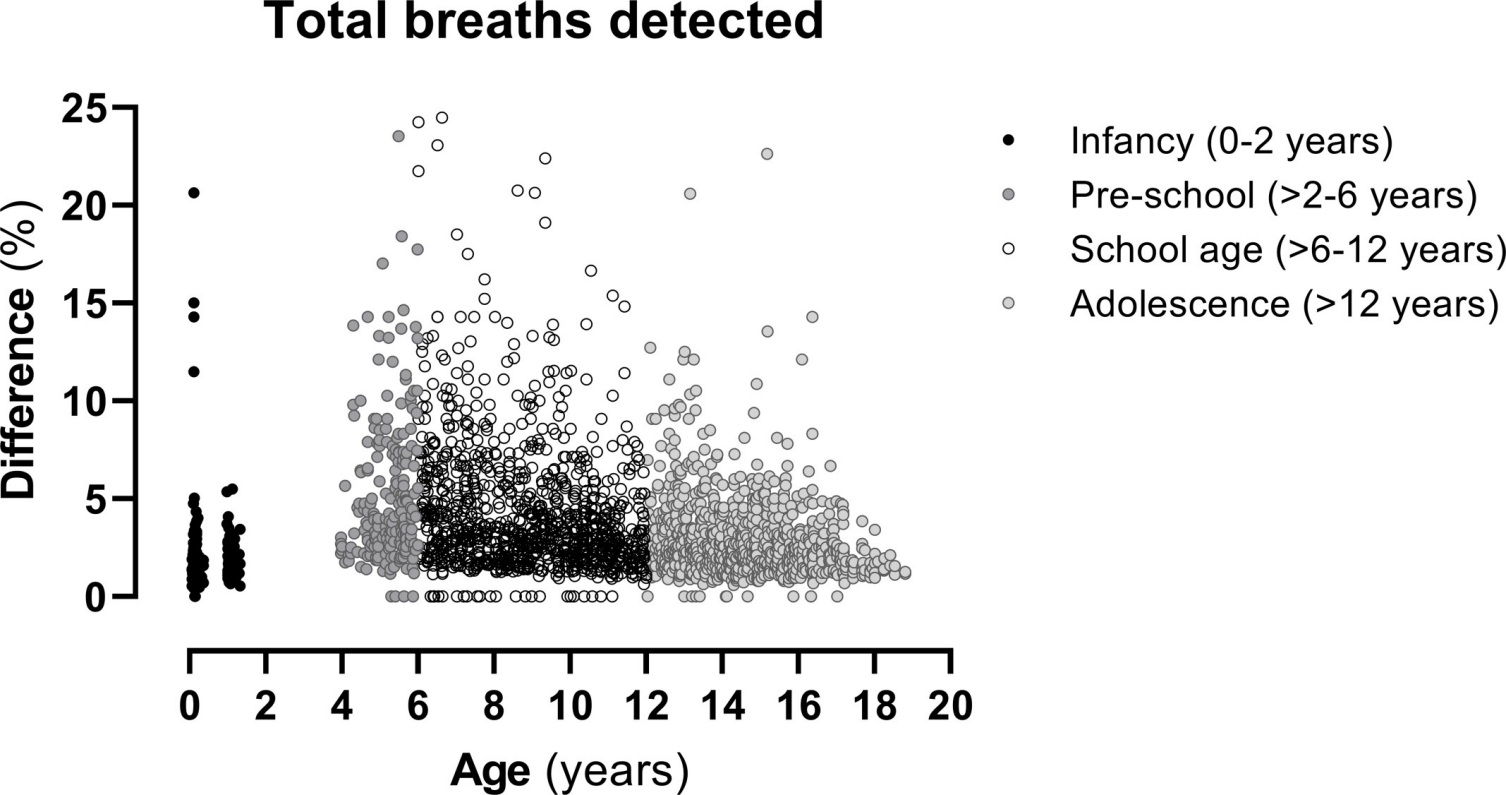
Relative difference [%] in total breaths detected. Comparison of the Spiroware and custom breath-dection algorithms by age group.

**Fig 3:**
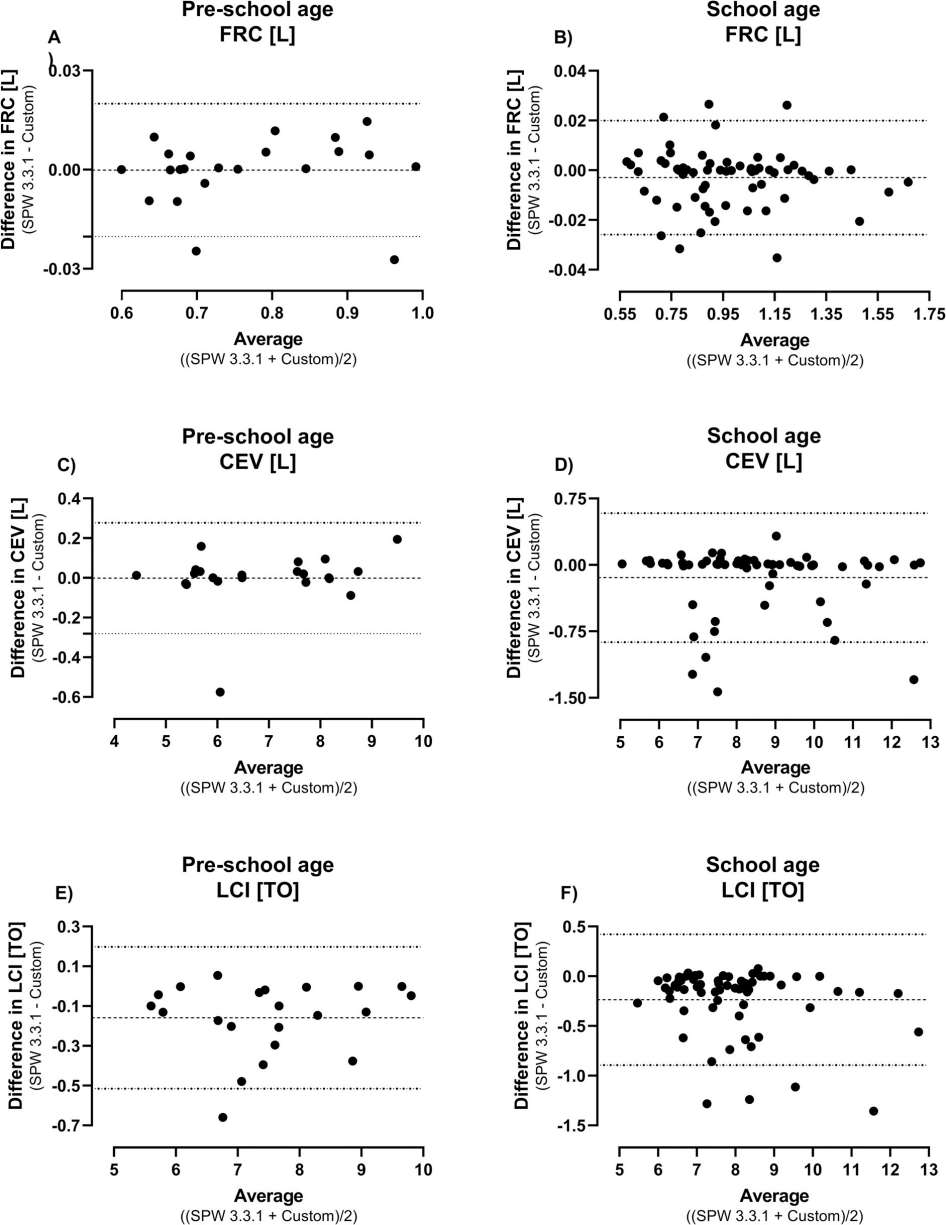
Bland-Altman plots of absolute difference between the commercial and custom breath detection algorithm. Shown are absolute differences in main MBW outcomes (FRC, CEV, LCI) for pre-school and school-age subjects with a difference in total breaths detected >10%. Abbreviations: FRC: functional residual capacity; CEV: cumulative expired volume; LCI: lung clearance index.

In trials with regular tidal breathing (e.g. infant MBW measurements), there were no considerable differences in outcomes (LCI, FRC, CEV) between the Spiroware and custom breath-detection algorithms. However, in 10% of pre-school and 6% of school-aged children, the number of breaths detected differed by more than 10% (Fig 2) with the commercial Spiroware software reporting on average more breaths. These differences in breaths caused a statistically significant increase in lung clearance index in pre-school (mean (SD) 3.1 (4.7) %; p = 0.007) and school aged children (mean (SD) 3.9 (8.0) %; p = 0.0003; Table 2 and Fig 3) when using the custom algorithm. As infants (at 6 weeks and 1 year of age) perform the test during sleep, there were only four measurements (1.2%) with a relevant difference in main MBW outcomes.

**Table 2:** Impact of breath-detection algorithms on main MBW outcomes. Relative difference (Spiroware–custom; mild (<5%), moderate (5 to 10%), high (>10%)) in main MBW outcomes (LCI, FRC, CEV) by age group (infants (0 to 2 years), pre-school age (>2 to 6 years), school age (>6 to 11 years), adolescents (>12 years)). Abbreviations: LCI_2.5%_: lung clearance index; FRC: functional residual capacity; CEV: cumulative expired volume.

Age group	Difference in breaths			Difference in LCI_2.5%_ [%]				Difference in FRC [%]				Difference in CEV [%]			
		n	n%	mean	SD	min	max	mean	SD	min	max	mean	SD	min	max
Infancy	mild	312	97.8	0.0	0.2	-3.8	0.0	0.0	0.2	-0.1	3.6	0.0	0.0	-0.1	0.3
	moderate	3	0.9	0.0	0.0	0.0	0.0	0.0	0.0	0.0	0.0	0.0	0.0	0.0	0.0
	high	4	1.3	-3.0	1.0	-4.0	-1.8	0.0	0.1	-0.2	0.2	0.3	0.1	0.2	0.4
Pre-school	mild	149	62.1	-0.5	2.4	-24.3	2.0	0.3	1.7	-2.5	20.3	-0.1	1.5	-15.3	0.8
	moderate	68	28.3	-1.1	1.7	-8.8	1.3	0.1	1.3	-3.4	4.6	-0.2	1.9	-8.7	4.2
	high	23	9.6	-3.1	4.7	-21.0	0.8	-0.2	1.5	-4.1	1.6	-0.9	4.6	-19.2	2.8
School age	mild	746	71.7	-0.2	1.8	-23.0	31.4	0.0	0.6	-6.7	6.9	-0.1	2.1	-30.7	36.0
	moderate	230	22.1	-0.8	3.0	-22.5	10.6	0.0	0.8	-6.8	3.4	-0.2	3.5	-28.0	13.2
	high	65	6.2	-3.9	8.0	-57.6	0.9	-0.5	2.1	-13.1	2.9	-3.0	10.6	-77.8	3.6
Adolescence	mild	974	89.1	-0.1	0.8	-16.7	8.4	0.0	0.2	-2.5	3.1	0.0	0.8	-18.8	8.0
	moderate	106	9.7	-0.6	2.0	-18.9	0.9	-0.1	0.6	-3.3	1.4	-0.4	2.3	-21.0	0.9
	high	13	1.2	-2.4	4.8	-17.6	0.0	-0.2	0.5	-1.5	0.8	-1.7	5.0	-16.8	0.9

### Rejected raw data in Spiroware

In 1960/2374 (82.6%) N_2_-MBW and 103/319 (32.3%) SF_6_-MBW trials, the commercial Spiroware breath detection algorithm rejected parts of tidal breaths and did not use them for the calculation of MBW outcomes (incomplete breaths before the first valid inspiration and after the last valid expiration are not considered in this analysis). While rejected parts of breaths and reclassified zero crossings resulted in a loss of raw data, the underlying conditions of the Spiroware algorithm resulted in a greater number of total breaths. On average, we found three or more occurrences per N_2_-MBW trial where data between expirations and inspirations (63.4%) or inspirations and expirations (52.5%) were discarded (Table 3). There were also discarded sections between two expirations or two inspirations in 38.3% and 25.9% of N_2_-MBW trials, respectively. We found considerably fewer rejections among infant SF_6_-MBW data (Table 3).

**Table 3:** Rejected data by Spiroware software. Occurrence: number of rejected sections per trial; zero crossings: number of affected zero crossings per trial; exp: expiration; insp: inspiration.

	N_2_					SF_6_				
	Occurence		Zero crossings			Occurence		Zero crossings		
Case	n	%	mean	SD	max	n	%	mean	SD	max
exp, invalid breathing, exp	910	38.3	3.8	4.9	45	39	12.2	8.0	14.2	77
insp, invalid breathing, insp	616	25.9	3.5	4.7	49	47	14.7	3.9	8.6	59
exp, invalid breathing, insp	1506	63.4	9.5	11.6	106	31	9.7	7.0	8.1	30
insp, invalid breathing, exp	1247	52.5	9.2	12.3	134	40	12.5	6.2	5.9	28
Total	1960	82.6				103	32.2			

Raw data rejection had a substantial effect on LCI. For data rejected between an expiration and an inspiration, LCI differed by mean (SD) 0.7 (2.5) TO (p<0.001) and for data rejected between an inspiration and an expiration, the LCI differed by 0.8 (2.9) TO (p<0.001).

### Agreement between the Horáček and custom breath-detection algorithm

In contrast to the comparison of the commercial Spiroware and custom breath-detection algorithms, the Horáček and custom algorithms obtained almost similar breath counts (mean (SD) 0.7 (0.9) breaths, p<0.001; S2 Fig) and this difference had minimal effects on MBW outcomes (S2 Table). Compared to the custom algorithm, we found that the discrimination between inspiration and expiration in the Horáček algorithm was at times inaccurate (S3 Fig).

## Discussion

The objective of this study was to investigate differences between a commercial and novel breath-detection algorithm and to characterize effects on MBW outcomes in children. We report considerable differences in MBW breath numbers between algorithms in preschool and school-age children with irregular breathing patterns. This led to an underestimation of ventilation inhomogeneity outcomes by the Spiroware algorithm, and depending on the child’s age and breathing pattern, the LCI changed by up to 1.7 TO (21%) in preschool and 4.7 TO (58%) in school-age children. There were no significant differences in breath number or MBW outcomes in sleeping infants or older children with more regular tidal breathing. The Spiroware breath-detection algorithm rejected raw data and/or re-classified zero-crossings so that while raw data was rejected, a greater number of breaths was reported.

We developed a custom breath detection algorithm for MBW measurements and tested it on a large dataset consisting of both nitrogen and sulfur hexafluoride MBW measurements across the entire paediatric age range. Our algorithm requires synchronized flow and carbon dioxide signals, and expirations are identified by increasing concentrations of measured CO_2_ above a threshold of 2% at the sensor. In regular tidal breathing, a breath-end corresponds to the time point when the flow changes its direction from negative to positive. However, variable and irregular breathing patterns may cause many zero crossings in the flow signal without actual gas exchange happening. The addition of a minimum volume criterion (for the volume of inspirations and expirations) has the potential to determine appropriate intervals for zero crossings but relies on pre-defined volume thresholds. However, an increase or peak in the synchronized CO_2_ signal enables the identification of expirations independent of additional (e.g. body-weight related) thresholds. Thus, the two components i) flow reversal, and ii) presence of CO_2_ enable a simple and reliable identification of inspirations and expirations.

Previous breath detection algorithms that made use of the CO_2_ concentration were designed for the bedside analysis of breathing patterns of patients receiving intensive care [18], the surveillance of obstructive sleep apnoea patients equipped with continuous positive airway pressure (CPAP) facemasks [19], and recently the analysis of distorted breathing patterns in MBW measurements [10]. The algorithm developed by Brunner et al. in 1985 did not include a minimum volume criterion and had fixed CO_2_ cutoffs of zero percent during inspirations and above zero percent during expirations [18]. However, during the prephase of MBW measurements (before the start of the washout), the subject breathes ambient room air where during inspiration an atmospheric CO_2_ concentration of 0.04% can be expected [20]. In addition, even slight delays in signal synchronization may influence the CO_2_ concentration to an extent where zero percent may not be reached [8]. During expirations, the CO_2_ concentration should reach 2% or more as an accumulation of CO_2_ may cause hypercapnia [21]. Therefore, we applied a single threshold of 2% to discriminate between inspirations and expirations.

The algorithm described by Horáček et al. improved the thresholds for CO_2_ (>2% for expirations and <0.5% for inspirations) and added a volume ratio as well as grouping of zero crossings [10]. However, we found that the discrimination between inspiration and expiration was at times inaccurate, most likely due to the mandatory volume ratio. A complete comparison between the Horáček and custom breath-detection algorithms is provided in the S1 File.

To the best of our knowledge, this is the first study to describe and characterize the rejection of raw MBW data by breath detection algorithms during the cumulative analysis of MBW outcomes. While our custom breath detection algorithm does not exclude raw data (segments of discarded zero crossings are added to the previous interval), the Spiroware algorithm rejects raw data, which in some cases even results in additional breaths (e.g. case 1 (exp, invalid breathing, exp) where the invalid breathing is kept and re-classified as an inspiration). This finding raises the important question of which MBW results should be calculated based on all breaths and which require additional criteria or selection. As recommended [8], indices of ventilation distribution should be based on all points of the washout curve (as these are less susceptible to measurement error than a single time point), and there should ideally be no data rejection during the critical period (10 breaths before achieving equilibrium and troughout the washout) that is used for the cumulative analysis (e.g., CEV, FRC, and LCI). However, tidal breathing parameters and indices based on single breaths (e.g. single breath washout or volumetric capnography) are less prone to error by breath detection algorithms as their analysis does not rely on the change of multiple signals over time [2].

There are very limited data on breath detection algorithms used in commercial MBW analysis software and their effect on MBW outcomes. While previous studies compared new algorithms to human experts as a reference, it was also reported that the experts themselves did not achieve identical breath counts [10, 18]. We therefore compared our custom breath detection algorithm on over 168,500 breaths with the built in Spiroware algorithm which is already in widespread use.

Over the years, the MBW technique has undergone continuous development which resulted in improved data analysis, updated analysis software and consequently changes in MBW outcomes. We recently identified and characterized a substantial sensor-crosstalk error in the Exhalyzer D device (Eco Medics AG, Duernten, Switzerland) that causes an overestimation of expired tracer gas concentrations, and consequently MBW outcomes [17]. A correction for this error was developed in collaboration with the manufacturer and is now available as a software update (Spiroware 3.3.1, Eco Medics AG, Duernten, Switzerland). In this study, we show that also breath detection algorithms can influence MBW outcomes significantly. Depending on the child’s age and breathing pattern, the LCI changed by up to 21% in preschool and 58% in school-age children, thus exceeding the recently proposed threshold between test occasions of 15% for preschool children during a period when spirometry may not yet be reliably performed or when FEV_1_ may still be normal [22]. Manufacturers should ensure that the underlying signal processing (including breath detection algorithms) is accurate and provide transparency relating to any deletion of raw data.

Our study represents a comprehensive examination of breath detection algorithms and their effects on MBW outcomes. We developed a novel breath detection algorithm as a possible alternative to current algorithms which reject data from the cumulative analysis of nitrogen and sulfur hexafluoride washouts or misinterpret inspirations and expirations. We did not perform a comparison to human experts as previous studies repeatedly reported a lack of agreement between examiners and subsequently between examiners and computerized algorithms [10, 19]. Further, we focused our analysis to MBW data gathered with the most common setup for nitrogen MBW in clinical trials (Exalyzer D/Spiroware setup), and therefore the results may not be generalizable to other devices.

The identification of breath ends and the discrimination between inspirations and expirations remains a challenging task, especially in measurements with irregular breathing. However, the analysis of MBW outcomes relies on the cumulative analysis of all washout breaths and the rejection of breaths should be limited. In an era where manufacturers develop devices for a variety of pulmonary function tests, breath detection algorithms based on flow reversal and the presence of CO_2_ could be used as an alternative to current methods.

## Conclusion

Accurate breath detection is challenging in young children. As the MBW technique relies on the cumulative analysis of all washout breaths, the rejection of breaths should be limited. Breath detection algorithms based on flow reversal and presence of CO_2_ could be used as an alternative to current methods.

## Supporting information

S1 Fig Flowdiagram of included raw data files. Abbreviations: N_2_: nitrogen; SF_6_: sulfur hexafluoride.
(PDF)

S2 Fig Relative difference [%] in total breaths detected. Comparison of the Horáček and custom breath-dection algorithms by age group.
(PDF)

S3 Fig Differences in the discrimination between inspirations and expirations between the Spiroware, Horáček, and custom breath-detection algorithms. Flow signals [m^3^/s] of the Spiroware 3.3.1 (A), Horáček (B), and custom (C) breath detection algorithm, with the corresponding CO_2_-signal (D) after signal processing (ATP correction, dynamic delay correction, BTPS correction, signal filtering, cross-talk-correction, and drift correction). Inspiration (green), expirations (blue), and rejected breaths (red) are shown.
(PDF)

S1 Table Agreement of LungSim and Spiroware MBW outcomes. MBW raw data gathered with the Exhalyzer D (Eco Medics AG, Duernten, Switzerland) was analyzed with Spiroware and LungSim analysis software. A-Files with a relative difference ≥0.1% underwent further investigation. Abbreviations: N_2_: nitrogen; SF_6_: sulfur hexafluoride; FRC: functional residual capacity; LCI: lung clearance index; SPW: Spiroware analysis software; LS: LungSim analysis software; MM_ss_: sidestream molar mass signal.
(PDF)

S2 Table Impact of breath-detection algorithms. Relative difference (Horáček–custom; mild (<5%), moderate (5 to 10%), high (>10%)) in main MBW outcomes (LCI, FRC, CEV) by age group (infants (0 to 2 years), pre-school age (>2 to 6 years), school age (>6 to 11 years), adolescents (>12 years)). Abbreviations: LCI_2.5%_: lung clearance index; FRC: functional residual capacity; CEV: cumulative expired volume.
(PDF)

S1 File (DOCX)

## References

[1] Subbarao P, Milla C, Aurora P, Davies JC, Davis SD, Hall GL, et al. Multiple-Breath Washout as a Lung Function Test in Cystic Fibrosis. A Cystic Fibrosis Foundation Workshop Report. Annals of the American Thoracic Society. 2015;12(6):932–9. doi: 10.1513/AnnalsATS.201501-021FR 26075554 PMC5466249

[2] Robinson PD, Goldman MD, Gustafsson PM. Inert gas washout: theoretical background and clinical utility in respiratory disease. Respiration; international review of thoracic diseases. 2009;78(3):339–55. doi: 10.1159/000225373 19521061

[3] Perrem L, Stanojevic S, Shaw M, Jensen R, McDonald N, Isaac SM, et al. Lung Clearance Index to Track Acute Respiratory Events in School-age Children with Cystic Fibrosis. Am J Respir Crit Care Med. 2020. 10.1164/rccm.202006-2433OC 33030967

[4] Rayment JH, Stanojevic S, Davis SD, Retsch-Bogart G, Ratjen F. Lung clearance index to monitor treatment response in pulmonary exacerbations in preschool children with cystic fibrosis. Thorax. 2018;73(5):451–8. doi: 10.1136/thoraxjnl-2017-210979 29449440

[5] Ramsey KA, Foong RE, Grdosic J, Harper A, Skoric B, Clem C, et al. Multiple-Breath Washout Outcomes Are Sensitive to Inflammation and Infection in Children with Cystic Fibrosis. Annals of the American Thoracic Society. 2017;14(9):1436–42. doi: 10.1513/AnnalsATS.201611-935OC 28481640

[6] Ramsey KA, Rosenow T, Turkovic L, Skoric B, Banton G, Adams A-M, et al. Lung Clearance Index and Structural Lung Disease on Computed Tomography in Early Cystic Fibrosis. Am J Respir Crit Care Med. 2016;193(1):60–7. doi: 10.1164/rccm.201507-1409OC 26359952

[7] Wielpütz MO, Puderbach M, Kopp-Schneider A, Stahl M, Fritzsching E, Sommerburg O, et al. Magnetic Resonance Imaging Detects Changes in Structure and Perfusion, and Response to Therapy in Early Cystic Fibrosis Lung Disease. Am J Respir Crit Care Med. 2014;189(8):956–65. doi: 10.1164/rccm.201309-1659OC 24564281

[8] Robinson PD, Latzin P, Verbanck S, Hall GL, Horsley A, Gappa M, et al. Consensus statement for inert gas washout measurement using multiple- and single- breath tests. Eur Respir J. 2013;41(3):507–22. doi: 10.1183/09031936.00069712 23397305

[9] Robinson PD, Latzin P, Ramsey KA, Stanojevic S, Aurora P, Davis SD, et al. Preschool Multiple-Breath Washout Testing. An Official American Thoracic Society Technical Statement. Am J Respir Crit Care Med. 2018;197(5):e1–e19. doi: 10.1164/rccm.201801-0074ST 29493315

[10] Horáček J, Koucký V, Hladík M. Novel approach to computerized breath detection in lung function diagnostics. Comput Biol Med. 2018;101:1–6. doi: 10.1016/j.compbiomed.2018.07.017 30081237

[11] Frauchiger BS, Carlens J, Herger A, Moeller A, Latzin P, Ramsey KA. Multiple breath washout quality control in the clinical setting. Pediatr Pulmonol. 2021;56(1):105–12. doi: 10.1002/ppul.25119 33058570

[12] EcoMedics. Operator’s Manual Exhalyzer D Pulmonary Function Testing Device. Duernten, Switzerland 2020.

[13] Fuchs O, Latzin P, Kuehni CE, Frey U. Cohort profile: the Bern infant lung development cohort. Int J Epidemiol. 2012;41(2):366–76. doi: 10.1093/ije/dyq239 21233140 PMC7108546

[14] Frauchiger BS, Binggeli S, Yammine S, Spycher B, Krüger L, Ramsey KA, et al. Longitudinal Course of Clinical Lung Clearance Index in Children with Cystic Fibrosis. Eur Respir J. 2020:2002686. 10.1183/13993003.02686-2020 33361098

[15] Korten I, Kieninger E, Yammine S, Regamey N, Nyilas S, Ramsey K, et al. The Swiss Cystic Fibrosis Infant Lung Development (SCILD) cohort. Swiss Med Wkly. 2018;148:w14618. doi: 10.4414/smw.2018.14618 29698544

[16] Gustafsson PM, Robinson PD, Lindblad A, Oberli D. Novel methodology to perform sulfur hexafluoride (SF6)-based multiple-breath wash-in and washout in infants using current commercially available equipment. Journal of applied physiology (Bethesda, Md: 1985). 2016;121(5):1087–97. doi: 10.1152/japplphysiol.00115.2016 27493195

[17] Wyler F, Oestreich MA, Frauchiger BS, Ramsey KA, Latzin P. Correction of sensor crosstalk error in Exhalyzer D multiple-breath washout device significantly impacts outcomes in children with cystic fibrosis. Journal of applied physiology (Bethesda, Md: 1985). 2021;131(3):1148–56. doi: 10.1152/japplphysiol.00338.2021 34351818

[18] Brunner J, Wolff G, Langenstein H, Cumming G. Reliable detection of inspiration and expiration by computer. 1985;1(4):221–6. 10.1007/BF01720186 3836286

[19] Nguyen CD, Amatoury J, Carberry JC, Eckert DJ. An automated and reliable method for breath detection during variable mask pressures in awake and sleeping humans. PLoS One. 2017;12(6):e0179030. doi: 10.1371/journal.pone.0179030 28609480 PMC5469467

[20] Hall BD, Crotwell AM, Kitzis DR, Mefford T, Miller BR, Schibig MF, et al. Revision of the World Meteorological Organization Global Atmosphere Watch (WMO/GAW) CO&lt;sub&gt;2&lt;/sub&gt; calibration scale. Atmospheric Measurement Techniques. 2021;14(4):3015–32.

[21] Shigemura M, Homma T, Sznajder JI. Hypercapnia: An Aggravating Factor in Asthma. Journal of Clinical Medicine. 2020;9(10):3207. doi: 10.3390/jcm9103207 33027886 PMC7599850

[22] Oude Engberink E, Ratjen F, Davis SD, Retsch-Bogart G, Amin R, Stanojevic S. Inter-test reproducibility of the lung clearance index measured by multiple breath washout. Eur Respir J. 2017;50(4):1700433. doi: 10.1183/13993003.00433-2017 28982773 PMC5898949

