# Supplementary figures and images for: Breath detection algorithms affect multiple-breath washout outcomes in pre-school and school age children
Source: PLoS One. 2022 Oct 14;17(10):e0275866. doi: 10.1371/journal.pone.0275866 (PMC9565421; doi:10.1371/journal.pone.0275866)

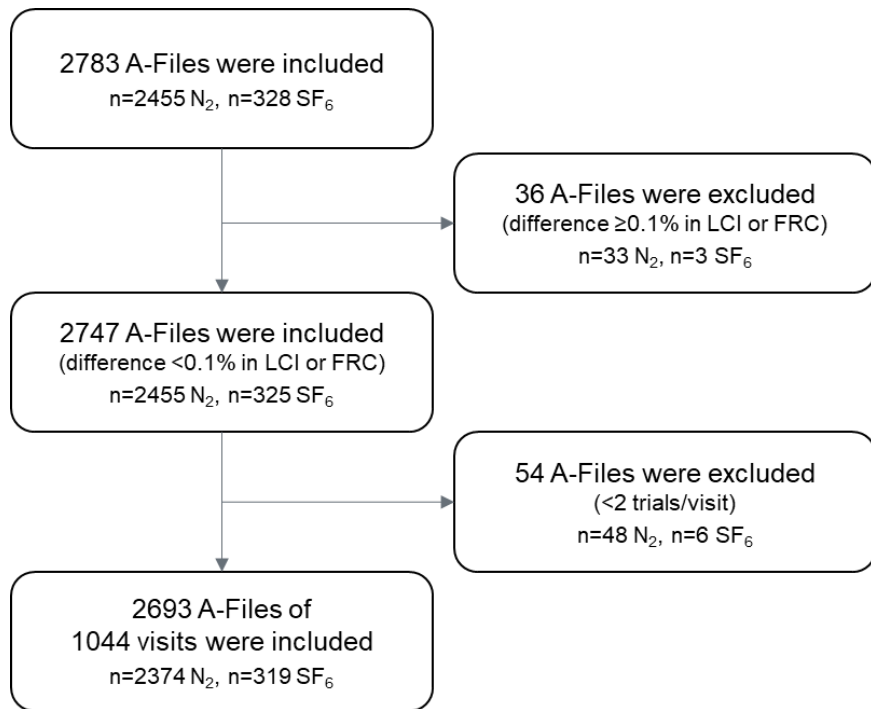

Supplement: S1 Fig — Abbreviations: N2: nitrogen; SF6: sulfur hexafluoride. (PDF) [file pone.0275866.s001.pdf]

Difference in total breaths detected

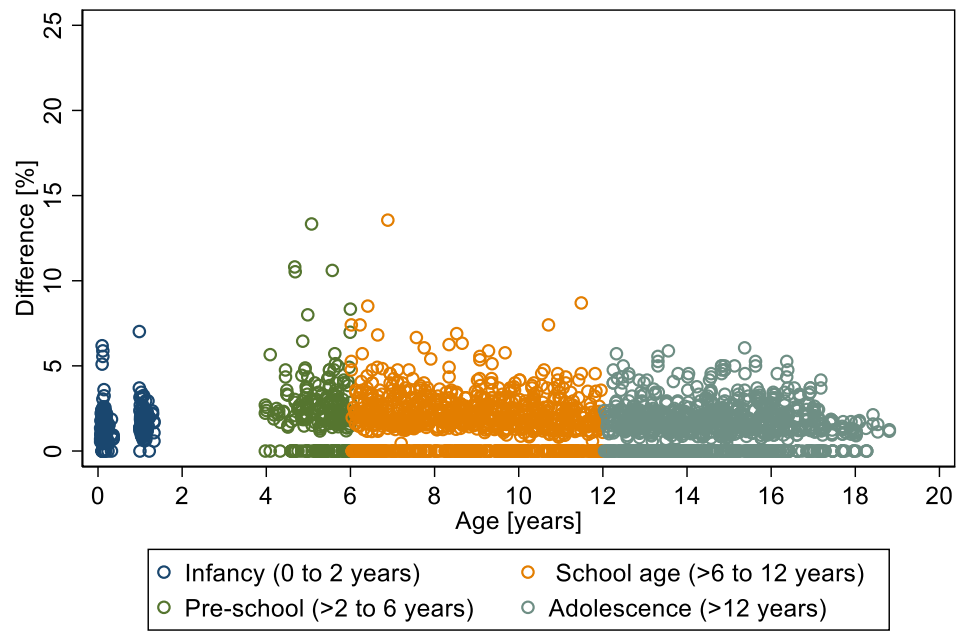

Supplement: S2 Fig — Comparison of the Horáček and custom breath-dection algorithms by age group. (PDF) [file pone.0275866.s002.pdf]

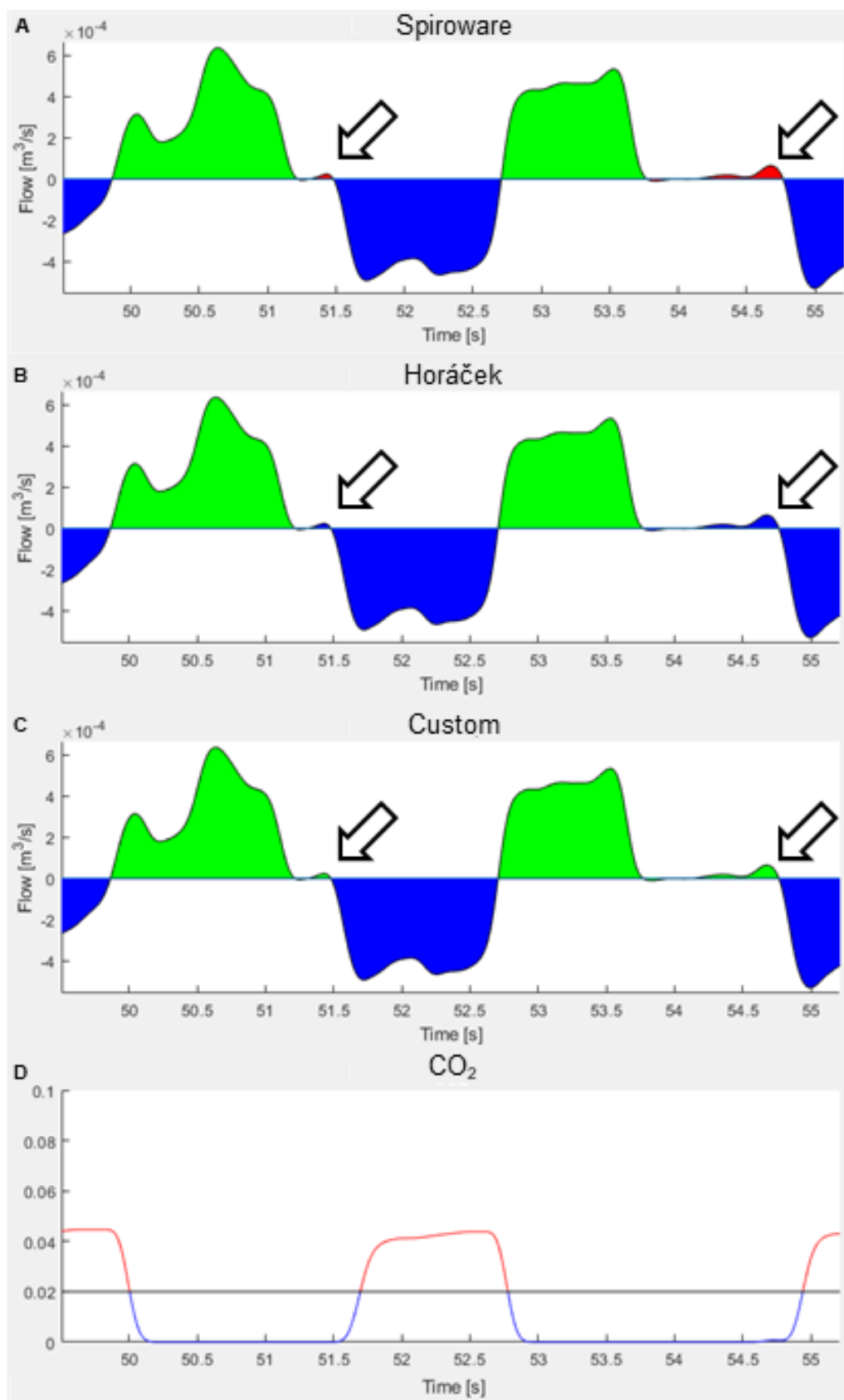

Supplement: S3 Fig — Flow signals [m3/s] of the Spiroware 3.3.1 (A), Horáček (B), and custom (C) breath detection algorithm, with the corresponding CO2-signal (D) after signal processing (ATP correction, dynamic delay correction, BTPS correction, signal filtering, cross-talk-correction, and drift correction). Inspiration (green), expirations (blue), and rejected breaths (red) are shown. (PDF) [file pone.0275866.s003.pdf]
