# Supplementary material for: Breath detection algorithms affect multiple-breath washout outcomes in pre-school and school age children
Source: PLoS One. 2022 Oct 14;17(10):e0275866. doi: 10.1371/journal.pone.0275866 (PMC9565421; doi:10.1371/journal.pone.0275866)
Supplement: S1 Table — MBW raw data gathered with the Exhalyzer D (Eco Medics AG, Duernten, Switzerland) was analyzed with Spiroware and LungSim analysis software. A-Files with a relative difference ≥0.1% underwent further investigation. Abbreviations: N2: nitrogen; SF6: sulfur hexafluoride; FRC: functional residual capacity; LCI: lung clearance index; SPW: Spiroware analysis software; LS: LungSim analysis software; MMss: sidestream molar mass signal. (PDF) [file pone.0275866.s004.pdf]

			Relative Error		Total	
**Tracer gas**	**Outcome**	**Diagnosis**	<0.1% [n]	≥0.1% [n]	n	%
Nitrogen	FRC [L]	Match >99.9%	2419	0	2419	98.5
		[SPW] Test-end criterion	2	10	12	0.5
		[SPW] Breath detection	0	1	1	0.0
		[LS] Breath detection	0	2	2	0.1
		[LS] Drift correction	0	3	3	0.1
		[LS] Synchronization CO_2	2	0	2	0.1
		[LS] Synchronization O_2/CO_2	1	0	1	0.0
		[LS] Rounding error	1	6	7	0.3
		[LS] Phase detection	0	3	3	0.1
		[LS] Crosstalk correction	0	1	1	0.0
		unknown	0	4	4	0.2
			**2425**	**30**	**2455**	**100.0**
	$LCI_{2.5\%}$ [TO]	Match >99.9%	2419	0	2419	98.5
		[SPW] Test-end criterion	0	12	12	0.5
		[SPW] Breath detection	0	1	1	0.0
		[LS] Breath detection	0	2	2	0.1
		[LS] Drift correction	0	3	3	0.1
		[LS] Synchronization CO_2	2	0	2	0.1
		[LS] Synchronization O_2/CO_2	1	0	1	0.0
		[LS] Rounding error	0	7	7	0.3
		[LS] Phase detection	0	3	3	0.1
		[LS] Crosstalk correction	0	1	1	0.0
		unknown	4	0	4	0.2
			**2426**	**29**	**2455**	**100.0**
Sulfur hexafluoride	FRC [L]	Match >99.9%	325	0	325	99.1
		[SPW] Test-end criterion	1	0	1	0.3
		[LS] Synchronization MM_{ss}	0	1	1	0.3
		[LS] Rounding error	0	1	1	0.3
			**326**	**2**	**328**	**100.0**
	$LCI_{2.5\%}$ [TO]	Match >99.9%	325	0	325	99.1
		[SPW] Test-end criterion	0	1	1	0.3
		[LS] Synchronization MM_{ss}	0	1	1	0.3
		[LS] Rounding error	0	1	1	0.3
			**325**	**3**	**328**	**100.0**
