# Supplementary material for: Breath detection algorithms affect multiple-breath washout outcomes in pre-school and school age children
Source: PLoS One. 2022 Oct 14;17(10):e0275866. doi: 10.1371/journal.pone.0275866 (PMC9565421; doi:10.1371/journal.pone.0275866)
Supplement: S1 File — (DOCX) [file pone.0275866.s006.docx]

**Breath detection algorithms affect multiple-breath washout outcomes in pre-school and school age children**

**Online Supplement**

Marc-Alexander Oestreich^1,2^ (https://orcid.org/0000-0001-9641-3691)
Florian Wyler^1^ (https://orcid.org/0000-0002-1232-1392)
Bettina Frauchiger^1^, MD, PhD (https://orcid.org/0000-0002-9519-9328)
Philipp Latzin, MD, PhD^1^ (https://orcid.org/0000-0002-5239-1571)
Kathryn Ramsey, PhD^1*^ (https://orcid.org/0000-0003-4574-6917).

^1^Division of Paediatric Respiratory Medicine and Allergology, Department of Paediatrics, Inselspital, Bern University Hospital, University of Bern, Switzerland.

^2^Graduate School for Health Sciences, University of Bern, Switzerland.

**Corresponding author**:
Kathryn Ramsey, PhD
Inselspital
Bern University Hospital
Freiburgstrasse 15, CH-3010 Bern, Switzerland
Email: [kathryn.ramsey@extern.insel.ch](mailto:kathryn.ramsey@extern.insel.ch)

### **LungSim**

### We developed LungSim, a custom Python script, to allow fast (batch) reloading of Eco Medics multiple-breath washout (MBW) data acquired with the Exhalyzer D/Spiroware setup (Eco Medics AG, Duernten, Switzerland). LungSim performs a complete signal processing (ATP correction, dynamic delay correction, BTPS correction, filtering, cross-talk correction, breath detection, and drift correction) on Eco Medics raw data (A-Files to B-Files) as well as computing MBW results (test trial tables) and breath tables for the Spiroware analysis software versions 3.2.1, 3.2.2, and 3.3.1, while being fully transparent. This enables the investigation of alternative algorithms (e.g. breath detection) and their effects on MBW results.

### **Agreement between LungSim and Spiroware**

We included 2783 raw data files (A-Files) of nitrogen and suflur hexafluordie MBW measurements gathered in our center. Based on a matching to Spiroware MBW outcomes (LCI and FRC, supplemental table 1), trials with a relative error of 0.1% or greater where excluded from further analysis (supplemental figure 1).


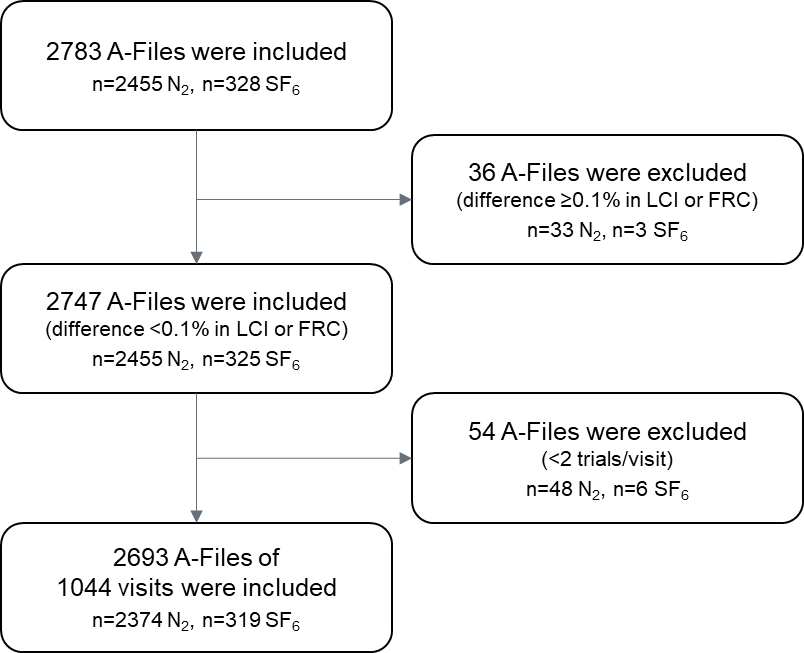


**S1 Fig. Flowdiagram of included raw data files**. Abbreviations: N_2_: nitrogen; SF_6_: sulfur hexafluoride.

All included raw data files (A-Files) could be analysed with LungSim. Overall, 2425/2455 (99%) of N_2_- and 325/328 (99%) of SF_6_-MBW files had similar MBW outcomes between Spiroware and LungSim (using the Spiroware breath detection algorithm; supplemental figure 1 and supplemental table 1).

**S1 Table. Agreement of LungSim and Spiroware MBW outcomes**. MBW raw data gathered with the Exhalyzer D (Eco Medics AG, Duernten, Switzerland) was analyzed with Spiroware (SPW) and LungSim (LS) analysis software. A-Files with a relative difference ≥0.1% underwent further investigation. Abbreviations: N_2_: nitrogen; SF_6_: sulfur hexafluoride; FRC: functional residual capacity; LCI: lung clearance index; SPW: Spiroware analysis software; LS: LungSim analysis software; MM_ss_: sidestream molar mass signal.

### **Alternative breath-detection algorithm (Horáček et al.)**

Horáček, Koucký, and Hladí recently proposed a novel breath-detection algorithm for the analysis of MBW data with severly distorted breathing patterns^1^. Once zero crossings in the flow signal are detected and numbered (1 to *N*), corresponding peaks in the CO_2_ signal are attributed and the volume for each inspiration (V_insp_) and expiration (V_exp_) is calculated. Next, a set of conditions apply: First, zero-crossings with insufficient CO_2_ concentration (<2% for expirations and >0.5% for inspirations) are discarded. Second, intervals are merged if the volume ratio of V_insp_ / V_exp_ between two intervals is greater five.

The analysis included 47 raw data files (A-files) from 19 pediatric patients (diagnosed with cystic fibrosis, primary ciliary diskenisia, or obstructive bronchitis). The files were analyzed using the Spiroware (v3.2.0) and Horáček breath-detection algorithms as well as by two specialists (reference standard). While the number of false positives was lower compared to the Spiroware algorithm and no false negatives were reported, the Horáček algorithm overestimated the total number of breaths in 33/47 files (70.2%).

### **Agreement between the Horáček and custom breath-detection algorithm**

In contrast to the comparison of the Spiroware and our custom breath-detection algorithm, the Horáček^1^ and custom algorithms obtained almost similar breath counts (mean (SD) 0.7 (0.9) breaths, p<0.001; supplemental Figure 2). The maximum difference was -8 breaths (in a trial with 59 breaths detected by Horáček) and 6 breaths (in a trial with 102 breaths detected by Horáček) in N_2_- and SF_6_-MBW measurements, respectively.

**S2 Fig. Relative difference [%] in total breaths detected.** Comparison of the Horáček and custom breath-dection algorithms by age group.

These differences in breath counts resulted in minimal effects on MBW outcomes (supplemental table 2).

**S2 Table. Impact of breath-detection algorithms.** Relative difference (Horáček – custom; mild (<5%), moderate (5 to 10%), high (>10%)) in main MBW outcomes (LCI, FRC, CEV) by age group (infants (0 to 2 years), pre-school age (>2 to 6 years), school age (>6 to 11 years), adolescents (>12 years)). Abbreviations: LCI_2.5%_: lung clearance index; FRC: functional residual capacity; CEV: cumulative expired volume.

Interestingly, we found that the discrimination between inspiration and expiration in the Horáček agorithm was at times inaccurate, most likely due to the volume ratio (supplemental figure 3).


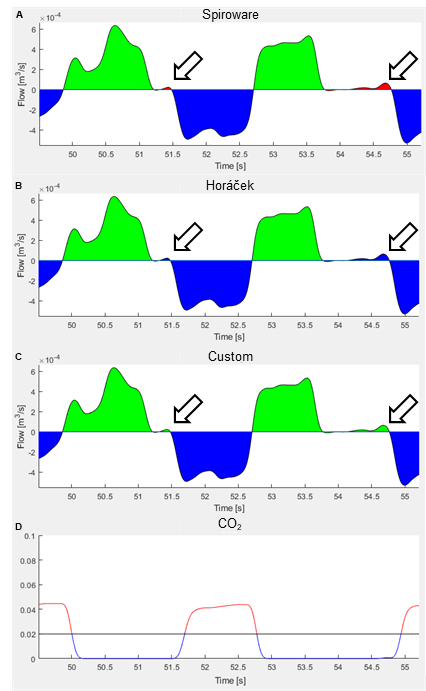


**S3 Fig.** **Differences in the discrimination between inspirations and expirations between the Spiroware, Horáček, and custom breath-detection algorithms.** Flow signals [m^3^/s] of the Spiroware 3.3.1 (A), Horáček (B), and custom (C) breath detection algorithm, with the corresponding CO_2_-signal (D) after signal processing (ATP correction, dynamic delay correction, BTPS correction, signal filtering, cross-talk-correction, and drift correction). Inspiration (green), expirations (blue), and rejected breaths (red) are shown.

**References**

1. Horáček J, Koucký V, Hladík M. 2018. Novel approach to computerized breath detection in lung function diagnostics. Comput Biol Med. 101:1–6.
